# Paving a way to treat spastic paraplegia 50

**DOI:** 10.1172/JCI170226

**Published:** 2023-05-15

**Authors:** Jonathan R. Brent, Han-Xiang Deng

**Affiliations:** The Ken and Ruth Davee Department of Neurology, Feinberg School of Medicine, Northwestern University, Chicago, Illinois, USA.

## Abstract

Spastic paraplegia 50 (SPG50) is a rare neurodegenerative disease caused by loss-of-function mutations in *AP4M1*. There are no effective treatments for SPG50 or any other type of SPG, and current treatments are limited to symptomatic management. In this issue of the *JCI*, Chen et al. provide promising data from preclinical studies that evaluated the efficacy and safety profiles of an AAV-mediated *AP4M1* gene replacement therapy for SPG50. AAV/*AP4M1* gene replacement partly rescued functional defects in SPG50 cellular and mouse models, with acceptable safety profiles in rodents and monkeys. This work represents a substantial advancement in therapeutic development of SPG50 treatments, establishing the criteria for taking AAV9/*AP4M1* gene therapy to clinical trials.

## Spastic paraplegia 50

Hereditary spastic paraplegia (HSP) is a heterogenous group of monogenic diseases characterized by upper motor neuron dysfunction and degeneration, leading to progressive spasticity of the lower limbs. HSP can present any time from infancy to adulthood. Clinically, HSP can present as a pure or complex form, with clinical signs being confined to lower limbs in pure HSP. Patients with complex HSP can have a variety of other symptoms, including ataxia, epilepsy, cognitive impairment, impaired vision, deafness, peripheral neuropathy, and others. HSP is genetically heterogenous with all classical modes of monogenic inheritance reported. To date, there are more than 100 genetic loci and 88 spastic paraplegia genes identified for different types of HSP (1). Based on the subcellular localization and putative function of the mutated proteins, it has become increasingly clear that HSP pathogenesis involves disruption of several cellular pathways, including axonal transport, membrane shaping and sorting, protein recycling/degradation, cytoskeletal dynamics, myelination, lipid metabolism, and mitochondrial function. Notably, therapeutic development for HSP largely lags behind its comprehensive genetic and mechanistic studies. There are no effective treatments to prevent, slow, or reverse HSP. Current treatment of HSP is limited to symptomatic relief (2).

Spastic paraplegia 50 (SPG50) is a rare and severe form of complex HSP. It is characterized by early infantile hypotonia, developmental delay, intellectual disability, and microcephaly. Over time, hypotonia evolves into spasticity of the lower limbs and then upper limbs, resulting in spastic tetraplegia. SPG50 is transmitted as an autosomal recessive disease caused by loss-of-function mutations in *AP4M1*, which encodes for the medium subunit of the adaptor protein complex 4 (AP-4) (3, 4). AP-4 is composed of four subunits, including two large chains (AP4B1 and AP4E1), a medium chain (AP4M1), and a small chain (AP4S1). Notably, biallelic loss-of-function mutations in each of these four AP-4 subunits lead to clinically overlapping HSP types, including SPG47 (*AP4B1*), SPG50 (*AP4M1*), SPG51 (*AP4E1*), and SPG52 (*AP4S1*), which could be collectively called “AP-4-associated HSP” or “AP-4 deficiency syndrome” (5–7). In mammals, there are five AP complexes (AP-1 to AP-5) that facilitate the selective sorting of transmembrane cargo proteins into vesicles and mediate related intracellular vesicle trafficking. AP-4 primarily localizes to the *trans-*Golgi network (TGN) and has been shown to mediate protein trafficking from the TGN to other cellular compartments, such as the endosomal-lysosomal system (8). Although the pathogenic mechanism by which loss of AP4M1 leads to SPG50 remains unclear, putting the missing *AP4M1* back into the affected cells is presumably the most rational option for SPG50 therapeutic development.

## Gene replacement therapy for SPG50

**Figure 1:**
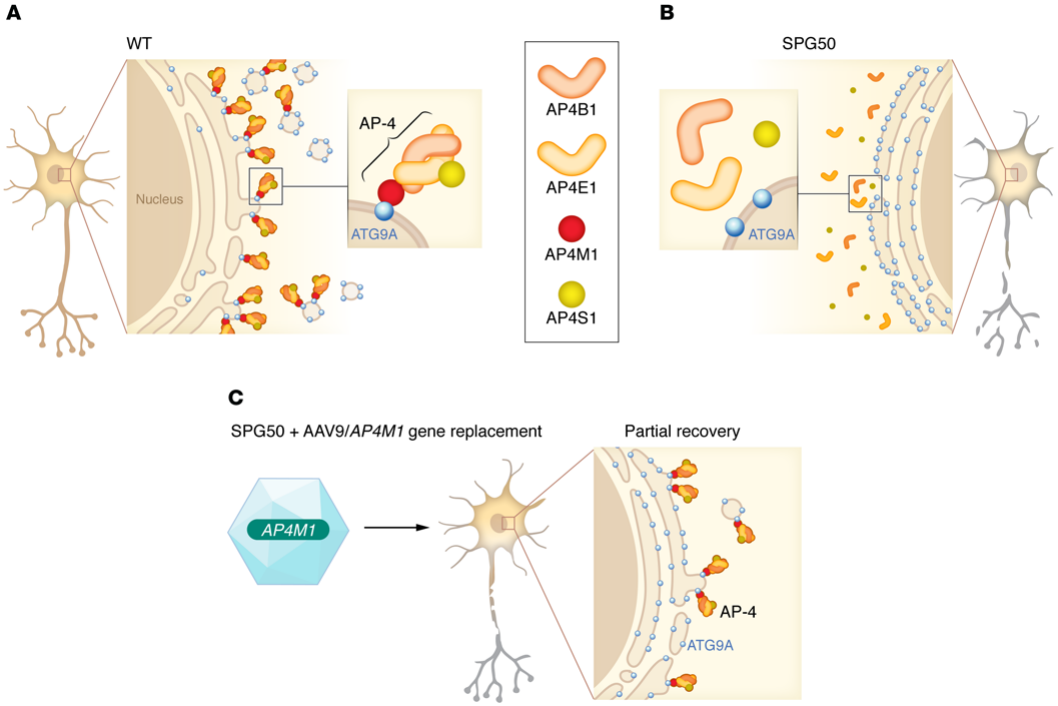
AAV/*AP4M1* gene therapy rescues AP4M1-deficient phenotypes. (**A**) AP-4 is composed of four subunits, including AP4B1, AP4E1, AP4M1, and AP4S1. The AP4M1 subunit recognizes and binds to specific cargo proteins, such as ATG9A, and facilitates vesicle budding from the *trans*-Golgi Network (TGN) to peripheral compartments.(**B**) Loss of AP4M1 prevents the formation of functional AP-4, leading to the retention of its cargo proteins on TGN, resulting in dysfunction and degeneration of neurons. (**C**) Chen et al. (9) show that AAV/*AP4M1* gene therapy can partly rescue functional defects in the SPG50 cellular and *Ap4m1*-KO animal models.

In this issue of the *JCI*, Chen et al. (9) provide promising data from preclinical studies that evaluated the efficacy and safety profiles of an AAV-mediated *AP4M1* gene replacement therapy for SPG50. The authors used a self-complementary AAV (scAAV) vector carrying the human *AP4M1* gene under the control of the ubiquitous synthetic promotor (UsP) (AAV/*AP4M1*). Transduction of the primary fibroblasts from patients with SPG50 with AAV2/*AP4M1* vector rescued the cellular phenotypes of AP4M1 deficiency, as shown by restoration of ATG9A trafficking from the TGN and by increased levels of AP4E1. To test the therapeutic potential in vivo, the authors administered AAV9/*AP4M1* to *Ap4m1*-KO mice by intrathecal injection. They observed dose-dependent increased levels of *AP4M1* mRNA in the brain regions, and partial restoration of the abnormal behavioral phenotypes, most robustly shown by the hind limb clasping test in the *Ap4m1*-KO mice (Figure 1). No apparent adverse effects were observed in the AAV9/*AP4M1*-treated *Ap4m1*-KO mice up to 20 months after injection.

The authors evaluated the safety profiles of the AAV9/*AP4M1* intrathecal administration further in normal mice, rats, and monkeys. They found that the AAV9/*AP4M1* gene therapy was overall safe and well tolerated in these animals. AAV9/*AP4M1* vector did not generate a detectable T cell immune response to either AAV9 or the human AP4M1 protein. Infrequent occurrence of hepatocellular adenomas was observed in some of the one-year-old mice, but this is unlikely related to the AAV9/*AP4M1* treatment. Minimal-to-mild neuronal toxicities were observed in rats and monkeys in three-month toxicology studies, which included neuronal degeneration noted microscopically in the lumbar dorsal root ganglion in both rats and monkeys. In the monkeys, neuronal toxicities were also observed in other regions of the central and peripheral nervous systems. Notably, these toxicities were dose related, as they preferentially occurred in the higher-dose cohorts, suggesting that dose optimization is required for the future clinical trials.

## Conclusions and implications

Chen et al. (9) have provided a comprehensive preclinical study that explores gene replacement as a therapeutic strategy for HSP. However, there are some intrinsic limitations. First, the *Ap4m1*-KO mice did not show robust and reliable motor phenotypes, even though patients with SPG50 eventually develop severe spastic tetraplegia. This inability to fully recapitulate the symptoms makes it challenging to effectively evaluate therapeutic efficacy. The improvement of phenotypes in Chen et al. (9) was primarily based on the hind limb clasping test. The improvements in other measures were variable, inconsistent with the doses, and thus difficult to interpret. Nevertheless, lacking robust motor phenotypes is not specific to the SPG50 mouse model, as no SPG mouse models to date display robust motor phenotypes, even though the degenerative pathology of the corticospinal tracts is prominent in the mice (10). This deficit may be partly due to the anatomical and functional differences of the corticospinal tracts between humans and rodents (11, 12). Second, dose-related neuronal toxicities, such as dorsal root ganglion degeneration, were observed in rats and monkeys, indicating the need for a lower dose range in clinical trials. Although a lower dose range will presumably reduce the toxicities, it may also limit the therapeutic efficacy. Enhancing *AP4M1* expression with a stronger promoter will likely reduce the AAV dose and thus minimize its related toxicities. The authors used UsP promoter to drive *AP4M1* expression. UsP is a short promoter (328 bp), which can be easily accommodated in the scAAV vector that has a size limit of approximately 2.3 kb. But UsP is a relatively weaker promoter, with about 15% activity of CMV promoter (13). Notably, a larger but stronger hybrid CMV enhancer/chicken β-actin promoter is used in a similar AAV9-based vector, called onasemnogene abeparvovec, for treating spinal muscular atrophy. But the much larger protein size of AP4M1 (453 amino acids) compared with that of SMN1 (262 amino acids) prevents the use of chicken β-actin or other much larger promoters to drive AP4M1 expression in the scAAV9 vector due to its size limit. Thus, the balance between therapeutic efficacy and toxicity may need further optimization in clinical trials.

The findings by Chen et al. (9) show promising evidence that AAV9/A*P4M1* gene replacement can partly rescue functional defects in the SPG50 cellular and mouse models with acceptable safety profiles in the rodents and monkeys when the dose is adjusted. Even though the authors state the need for further inquiry, the study by Chen et al. (9) provides critical preclinical data necessary to push AAV9/*AP4M1* gene therapy to clinical trials. This study represents a substantial advance in therapeutic development, particularly when considering the poor prognosis of SPG50.

## Version 1 05/15/2023

Electronic publication

## References

[1] Elsayed LEO (2021). Insights into clinical, genetic, and pathological aspects of hereditary spastic paraplegias: a comprehensive overview. Front Mol Biosci.

[2] Shribman S (2019). Hereditary spastic paraplegia: from diagnosis to emerging therapeutic approaches. Lancet Neurol.

[3] Verkerk AJ (2009). Mutation in the AP4M1 gene provides a model for neuroaxonal injury in cerebral palsy. Am J Hum Genet.

[4] Tuysuz B (2014). Autosomal recessive spastic tetraplegia caused by AP4M1 and AP4B1 gene mutation: expansion of the facial and neuroimaging features. Am J Med Genet A.

[6] Ebrahimi-Fakhari D (2020). Defining the clinical, molecular and imaging spectrum of adaptor protein complex 4-associated hereditary spastic paraplegia. Brain.

[7] Abou Jamra R (2011). Adaptor protein complex 4 deficiency causes severe autosomal-recessive intellectual disability, progressive spastic paraplegia, shy character, and short stature. Am J Hum Genet.

[8] Mattera R (2020). The role of AP-4 in cargo export from the trans-Golgi network and hereditary spastic paraplegia. Biochem Soc Trans.

[9] Chen X (2023). Intrathecal AAV9/*AP4M1* gene therapy for hereditary spastic paraplegia 50 shows safety and efficacy in preclinical studies. J Clin Invest.

[10] Deng H-X (2007). Distal axonopathy in an alsin-deficient mouse model. Hum Mol Genet.

[11] Muir GD, Whishaw IQ (1999). Complete locomotor recovery following corticospinal tract lesions: measurement of ground reaction forces during overground locomotion in rats. Behav Brain Res.

[12] Yang HW, Lemon RN (2003). An electron microscopic examination of the corticospinal projection to the cervical spinal cord in the rat: lack of evidence for cortico-motoneuronal synapses. Exp Brain Res.

[13] Karumuthil-Melethil S (2016). Novel vector design and hexosaminidase variant enabling self-complementary adeno-associated virus for the treatment of Tay-Sachs disease. Hum Gene Ther.

